# Genetic Polymorphisms in MHC Classes I and II Predict Outcomes in Metastatic Colorectal Cancer †

**DOI:** 10.3390/ijms26062556

**Published:** 2025-03-12

**Authors:** Pooja Mittal, Francesca Battaglin, Yan Yang, Shivani Soni, Sebastian Stintzing, Aparna R. Parikh, Karam Ashouri, Sandra Algaze, Priya Jayachandran, Lesly Torres-Gonzalez, Wu Zhang, Chiara Cremolini, Volker Heinemann, Joshua Millstein, Indrakant K. Singh, Heinz-Josef Lenz

**Affiliations:** 1 Division of Medical Oncology, Norris Comprehensive Cancer Center, Keck School of Medicine, University of Southern California, Los Angeles, CA 90089, USA; pm_344@usc.edu (P.M.);; 2 Department of Zoology, Deshbandhu College, University of Delhi, New Delhi 110019, India; 3 Department of Population and Public Health Sciences, Keck School of Medicine, University of Southern California, Los Angeles, CA 90032, USA; 4 Medical Department, Division of Oncology and Hematology, Charité Universitätsmedizin, 10117 Berlin, Germany; 5 Division of Hematology and Oncology, Massachusetts General Hospital, Boston, MA 02114, USA; 6 Department of Medical Oncology, University of Pisa, 56126 Pisa, Italy; 7 Department of Hematology/Oncology, LMU Klinikum, University of Munich, Comprehensive Cancer Center Munich, 81377 Munich, Germany; 8 Delhi School of Public Health, Institute of Eminence, University of Delhi, New Delhi 110007, India

**Keywords:** antigen presentation, colorectal cancer, MHC, biomarkers, survival

## Abstract

The immune system is alerted for virally infected cells in the body by the antigen presentation pathway, which is in turn mediated by the major histocompatibility complex (MHC) class I and II molecules. Cancer cells overcome immune evasion as a major hallmark by downregulation of the antigen presentation pathway. Therefore, the present study aimed to explore the effect of genetic variants in genes involved in MHC class I and II pathways in patients treated with first-line chemotherapy in combination with targeted antibodies in metastatic colorectal cancer (mCRC) patients. Genomic DNA from the blood samples of 775 patients enrolled in three independent, randomized, first-line trials, namely TRIBE (FOLFIRI-bevacizumab, N = 215), FIRE-3 (FOLFIRI-bevacizumab, N = 107; FOLFIRI-cetuximab, N = 129), and MAVERICC (FOLFIRI-bevacizumab, N = 163; FOLFOX6-bevacizumab, N = 161), was genotyped through OncoArray, a custom array manufactured by Illumina including approximately 530K SNP markers. The impact on the outcome of 40 selected SNPs in 22 genes of MHC class I and II pathways was analyzed. We identified several SNPs in multiple genes associated with targeted treatment benefits across different treatment arms in our study population (*p* < 0.05). Treatment–SNP interaction analyses confirmed a significant treatment interaction with the targeted agents (bevacizumab vs. cetuximab) and the chemotherapy backbone (FOLFIRI vs. FOLFOX) in certain selected SNPs. Our results highlight a potential role for MHC SNPs as prognostic and predictive biomarkers for first-line treatment in mCRC, with differential effects based on the biologic agent and chemotherapy backbone. These biomarkers, when further validated, may contribute to personalized treatment strategies for mCRC patients.

## 1. Introduction

Colorectal cancer (CRC) remains a leading cause of cancer-related mortality among both men and women. According to cancer statistics, an estimated 154,270 new CRC cases and 52,900 CRC-related deaths, including both sexes, will emerge in United States in the year 2025. Though CRC is the second-ranking cause of cancer deaths in both sexes combined, improved screening and treatment strategies have led to a sustained reduction in CRC-associated mortality in both men and women [1]. Biomarkers can help in early detection, disease progression, and treatment outcomes in CRC patients [2].

**Figure 1:**
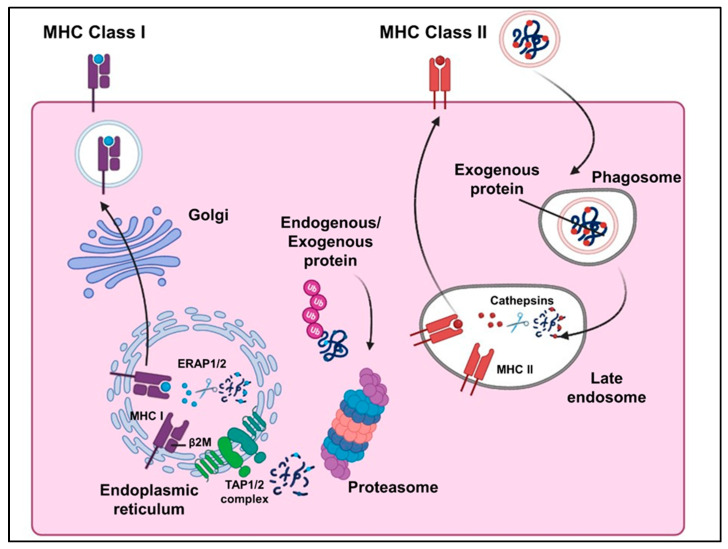
Schematic representation of the antigen processing and presentation pathway (MHC classes I and II).

The processing of antigens (antigen processing) and their presentation (antigen presentation) are two important steps of adaptive immune response. The glycoprotein receptors/proteins encoded by the major histocompatibility complex (MHC) are expressed on the surface of the antigen presenting cells (APCs) bound with ligands (processed antigens) and are recognized by T cells, which kill the cancer cells through TCR engagement with the neoantigen. The MHC can be divided into two major classes, MHC class I and II, which present processed and bound antigenic peptides to CD8+ T cells and CD4+ T cells, respectively [3]. MHC molecules are known as the Human Leukocyte Antigens (HLA) in humans, and the HLA genes are located on the short arm of chromosome 6. The HLA class I genes encode for the classical (HLA-A, -B, and -C) present endogenous/exogenous peptides processed by the proteasome to the CD8+ T Cells and non-classical (HLA-E, -F, -G) molecules, while the HLA class II encodes for molecules HLA-DR, -DP, -DQ, -DM, and -DO, which present exogenous peptides processed via lysosomal proteolysis to CD4+ helper T cells [4]. Other than the HLA complex, major genes involved in the antigen processing of MHC class I molecules include endoplasmic reticulum aminopeptidases (ERAP1 and ERAP2), transporters associated with antigen processing (TAP1 and TAP2), tapasin/TAP-binding protein (TAPBP), and beta-2-microglobulin (B2M) [3,5,6]. The *CIITA* gene encodes for the transcriptional activator that acts as a master switch for antigen presentation and regulates the expression of MHC class II genes [7] (Figure 1). MHC molecules are the major players in the adaptive immune response, and loss or mutations in MHC genes may increase the risk of infectious diseases or malignant/cancerous growths, hence they are among the most frequently mutated genes pan-cancer [3,8]. Cancer cells may express several tumor-specific or tumor-associated antigens and neoantigens, which can be recognized by the T cells and trigger an immune response. However, cancer cells can generally develop mechanisms to escape the host immune system and avoid immune destruction, including loss of function mutations in the genes associated with the antigen processing and presentation machinery (APM), which impairs MHC expression up to 93% in common cancers [9,10,11].

CRC is a heterogeneous disease characterized by genomic instability and immune cell infiltration which has been classified into several molecular subtypes based on gene expression signatures and activated pathways. T-cell-based immune therapies are emerging as promising treatment options for CRC and other malignancies [12]. Despite these advances, the prevalence and impact of SNPs and other mutations in MHC genes in relation to metastatic tumors remains largely elusive [8]. Strategies to activate the APM, including MHC gene expression in cancer, might increase anti-tumor immunity and thus may become an alternative approach for cancer treatment [11]. MHC class I and II genes are also associated with immune infiltration in the tumor microenvironment in metastatic CRC [13]. Studies have shown that the downregulation or loss of MHC class I expression correlates with poor prognosis in CRC, conferred by the avoidance of T-cell-mediated immunity. However, the downregulation of MHC class I expression sensitizes cells to natural killer (NK)-cell-mediated cytotoxicity via the loss of MHC class I inhibitory ligands and the activation of killer cell immunoglobulin-like receptors (KIRs). Interestingly, studies also hint towards the avoidance of this NK-cell-mediated clearance in tumor cells via the temporary and reversible upregulation/modulation of MHC class I expression via (post-) transcriptional modifications [14,15]. Previous studies highlight the association of single nucleotide polymorphisms (SNPs) in the APM and HLA genes with the anti-cancer response to chemotherapy, including non-small cell lung cancer (NSCLC) [16].

In the present study, we hypothesized that common and functional SNPs within MHC class I and II genes would correlate with survival outcomes in cetuximab- or bevacizumab-treated mCRC patients. To test our hypothesis, we used genetic and clinical data from three independent randomized clinical trials, FIRE-3 [17], TRIBE [18], and MAVERICC [19], comparing cetuximab vs. bevacizumab in combination with FOLFIRI/FOLFOX in the first-line treatment of patients with mCRC [20].

## 2. Results

### 2.1. Patient Characteristics

**Figure 2:**
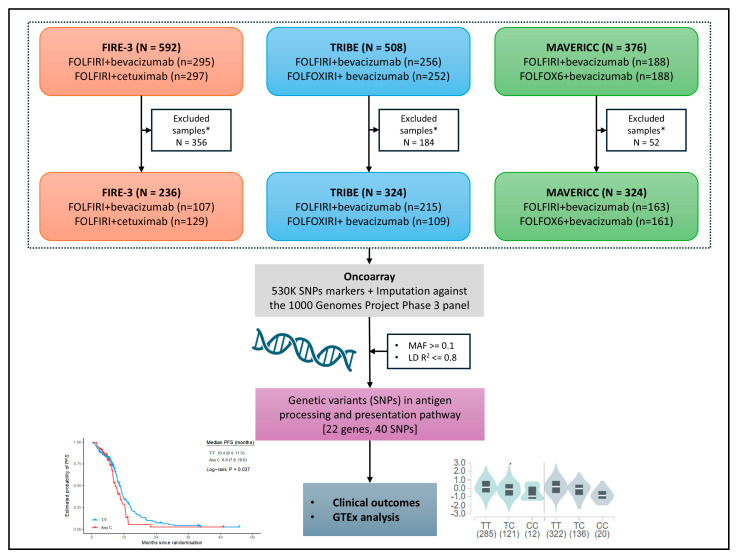
Consort diagram and workflow of the study. * Patients without sufficient peripheral whole blood samples, SNP data, and/or any oth-er relevant data for analyses were excluded from the study.

A total of 775 patients (enrolled in three independent, randomized, first-line trials) were included in the present study: 215 patients were in the bevacizumab cohort of TRIBE (FOLFIRI-bevacizumab), 107 patients were in the bevacizumab cohort of FIRE-3 (FOLFIRI-bevacizumab), 129 patients were in the cetuximab cohort of FIRE-3 (FOLFIRI-cetuximab), 163 patients were in the bevacizumab cohort (FOLFIRI-bevacizumab) of MAVERICC, and 161 patients were in the bevacizumab cohort (FOLFOX6-bevacizumab) of MAVERICC (Figure 2). The median follow-up time, PFS, and OS were 48.9, 9.7, and 26.2 months in TRIBE; 23.3, 12.5, and 27.4 months in MAVERICC; and 26.7, 11.5, and 31.4 months in the FIRE-3 FOLFIRI-bevacizumab cohort, respectively. The median follow-up time, PFS, and OS were 29.1, 12.8, and 49.8 months in the FOLFIRI-cetuximab cohort of FIRE-3, and 26.8, 10.1, and 24.7 months in FOLFOX6-bevacizumab cohort of MAVERICC, respectively.

### 2.2. Association of MHC Class I and II SNPs with Clinical Outcomes in the Cetuximab Cohort

**Figure 3:**
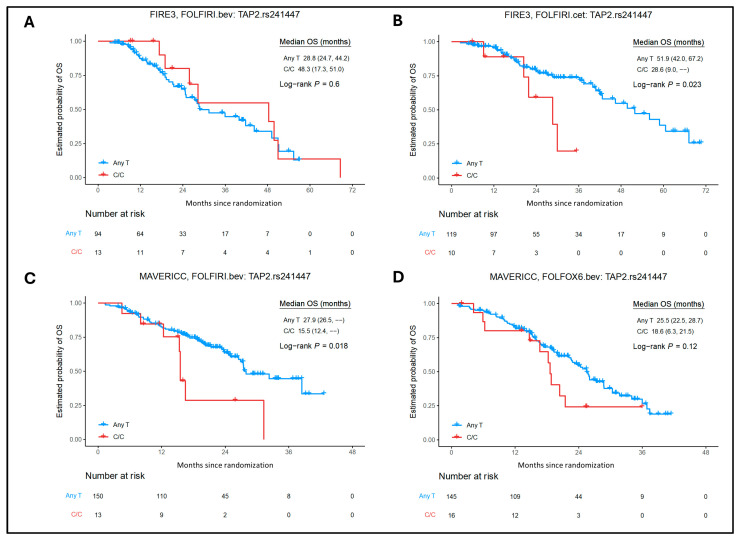
Association between *TAP2* rs241447 expression and patient outcomes in the FIRE-3 and MAVERICC trials. Kaplan–Meier curves show OS stratified by *TAP2* rs241447 alleles any T and C/C tumor expression according to different treatments of the FIRE-3 and MAVERICC trials. (**A**) FIRE-3 bev/FOLFIRI OS, (**B**) FIRE-3 cet/FOLFIRI OS, (**C**) MAVERICC bev/FOLFIRI OS, (**D**) MAVERICC bev/FOLFOX6 OS. Bev, bevacizumab; cet, cetuximab; OS, overall survival.

Among the 24 candidate SNPs for MHC class I genes, *TAP2* rs241447, *TAP2* rs1044043, *TAPBP* rs3106191, and *HLA-B* rs2770 showed significant associations with clinical outcomes in both uni- and multivariate analyses of the cetuximab cohort (Table S3). Patients with mCRC and *TAP2* rs241447 C/C allele (n = 10) showed significantly shorter OS than any T carriers (n = 119) [28.6 versus 51.9 months, hazard ratio (HR) = 2.91; 95% confidence interval (CI) = 1.11–7.67; *p* = 0.023] (Figure 3); *TAP2* rs1044043 A/A allele (n = 10) showed significantly better PFS than any C carriers (n = 119) [32.1 versus 12.1 months, HR = 0.47; 95% CI = 0.22–1.02; *p* = 0.045]; *TAPBP* rs3106191 any A allele (n = 66) showed significantly shorter PFS than C/C carriers (n = 63) [10.3 versus 15.1 months, HR = 1.75; 95% CI = 1.18–2.59); *p* = 0.0049]; *HLA-B* rs2770 any G allele (n = 67) showed significantly better OS than A/A carriers (n = 20) [56.1 versus 37.4 months, HR = 0.42; 95% CI = 0.19–0.93); *p* = 0.027] in univariate analysis. The abovementioned results were confirmed in multivariate analysis: *TAP2* rs241447 (C/C allele versus any T, HR = 3.35; 95% CI = 1.24–9.08; *p* = 0.035); *TAP2* rs1044043 (A/A allele versus any C, HR = 0.34; 95% CI = 0.15–0.78; *p* = 0.0038); *TAPBP* rs3106191 (any A allele versus C/C, HR = 1.86; 95% CI = 1.24–2.79; *p* = 0.0026); and *HLA-B* rs2770 (any G allele versus A/A, HR = 0.38; 95% CI = 0.16–0.86; *p* = 0.027). Apart from these, SNPs in the following genes showed significance only in univariate analysis: *TAP1* rs1135216 any C showed better OS compared with T/T (HR = 0.43; 95% CI = 0.19–0.99; *p* = 0.042); *TAP2* rs1800454 any T showed better OS compared with C/C (HR = 0.42; 95% CI = 0.17–0.99; *p* = 0.042); and *HLA-G* rs1610696 any G showed better PFS compared with C/C (HR = 0.67; 95% CI = 0.45–1.00; *p* = 0.046). *TAP2* rs1044043 (dominant model) any A allele showed better PFS compared with C/C carriers (HR = 0.62; 95% CI = 0.40–0.94; *p* = 0.021) in multivariate analysis.

Among the 16 candidate SNPs for MHC class II genes, *HLA-DPB1* rs3097671 and *HLA-DRA* rs7192 showed significant associations with clinical outcomes in both uni- and multivariate analyses in the cetuximab cohort. Patients with mCRC and *HLA-DPB1* rs3097671 any C allele (n = 38) showed significantly shorter PFS than G/G carriers (n = 91) [9.9 versus 13.5 months, HR = 1.69; 95% CI = 1.11–2.58; *p* = 0.014] and *HLA-DRA* rs7192 any T allele (n = 82) showed significantly better PFS [13.9 versus 8.7 months, HR = 0.62; 95% CI = 0.42–0.92; *p* = 0.014] and OS [56.1 versus 40.8 months, HR = 0.53; 95% CI = 0.29–0.98; *p* = 0.039] than G/G carriers (n = 47). The abovementioned results were confirmed in multivariate analysis, *HLA-DPB1* rs3097671 (any C versus G/G, HR = 1.76; 95% CI = 1.14–2.73; *p* = 0.014), and *HLA-DRA* rs7192 (any T versus G/G, HR = 0.65; 95% CI = 0.44–0.98; *p* = 0.043) for PFS and (any T versus G/G, HR = 0.52; 95% CI = 0.28–0.98; *p* = 0.044) for OS. Apart from these, *CIITA* rs4774 any C showed better OS compared with G/G (60.6 vs. 42.0 months, HR = 0.50; 95% CI = 0.25–0.98; *p* = 0.039); *HLA-DMB* rs10751 any A showed shorter PFS compared with G/G (9.5 vs. 12.9 months, HR = 1.76; 95% CI = 1.08–2.87; *p* = 0.03) in only multivariate analysis; and *HLA-DOB* rs11244 A/A showed shorter PFS compared with any G (7.8 vs. 12.8 months, HR = 2.18; 95% CI = 1.00–4.75; *p* = 0.045) in univariate analysis (Table S4).

Additionally, the multiple hypothesis testing corrections were computed and SNPs with *q* values < 0.1 were considered as statistically significant. *TAP2* rs1044043 and *TAPBP* rs3106191 both showed a *q* value < 0.1 for PFS in multivariate analysis in the FIRE-3 FOLFIRI-cetuximab cohort.

### 2.3. Association of MHC Class I and II SNPs with Clinical Outcomes in the Bevacizumab Cohorts

In the bevacizumab cohorts (FIRE-3, TRIBE, and MAVERICC), for MHC class I genes SNPs, there were no significant associations between *TAP1* rs1135216 and OS, *TAP2* rs1800454 and OS, *TAP2* rs1044043 and PFS, *TAPBP* rs3106191 and PFS, *HLA-B* rs2770 and OS, and *HLA-G* rs1610696 and PFS, in both univariate and multivariate analyses (Table S3). Similarly, for MHC class II genes SNPs, significant associations between *HLA-DMB* rs10751 and PFS, *HLA-DOB* rs11244 and PFS, *HLA-DPB1* rs3097671 and PFS, and *HLA-DRA* rs7192 and both PFS/OS were not found in either univariate or multivariate analyses. Patients carrying *TAP2* rs241447 C/C allele (n = 13) showed significantly shorter OS than any T carriers (n = 150) [15.5 versus 27.9 months, HR = 2.40, 95% CI = 1.13–5.09; *p* = 0.018] in univariate analysis, and the results were confirmed in multivariate analysis (HR = 2.87, 95% CI = 1.30–6.33; *p* = 0.019). Interestingly, patients carrying *CIITA* rs4774 had an opposite effect compared with the cetuximab cohort, with C/C allele (n = 10) showing shorter OS compared with any G carriers (n = 95) [20.1 versus 39.9 months, HR = 2.55, 95% CI = 1.13–5.77; *p* = 0.02] in univariate analysis (but not in multivariate analysis) (Table S4).

**Figure 4:**
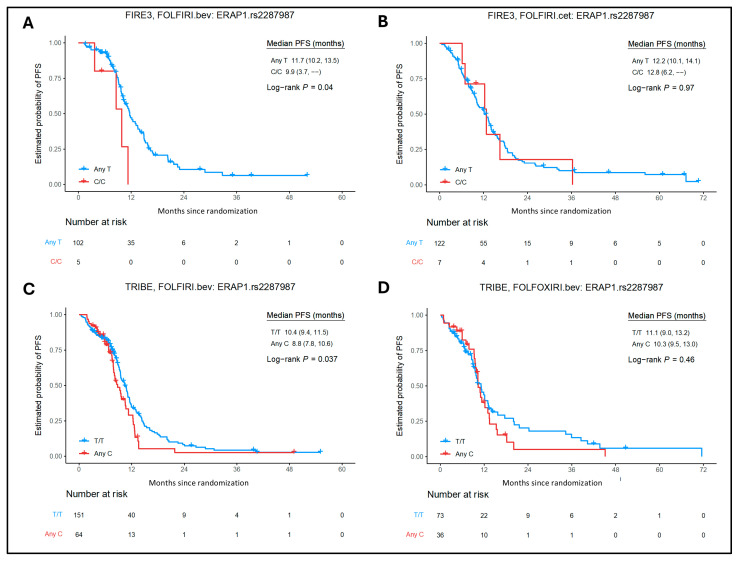
Association between *ERAP1* rs2287987 expression and patient outcomes in the FIRE-3 and TRIBE trials. Kaplan–Meier curves show PFS stratified by *ERAP1* rs2287987 alleles any T and C/C tumor expression according to different treatments of the FIRE-3 and TRIBE trials. (**A**) FIRE-3 bev/FOLFIRI PFS, (**B**) FIRE-3 cet/FOLFIRI PFS, (**C**) TRIBE bev/FOLFIRI PFS, (**D**) TRIBE bev/FOLFOXIRI PFS. Bev, bevacizumab; cet, cetuximab; PFS, progression-free survival.

Several significant associations between other SNPs and OS/PFS were found in the bevacizumab cohorts (Tables S3 and S4). In the FIRE-3 bevacizumab cohort, patients carrying *ERAP1* rs2287987 C/C allele (n = 5) showed worse PFS compared with any T carriers (n = 102) (9.9 versus 11.7 months) in both univariate (HR = 2.81, 95% CI = 1.00–7.89; *p* = 0.04) and multivariate (HR = 3.46, 95% CI = 1.18–10.12; *p* = 0.049) analyses (Figure 4), while *HLA-DMB* rs1042337 G/G carriers (n = 5) showed better PFS compared with any A allele (n = 102) in multivariate analysis [21.7 versus 11.4 months, HR = 0.16, 95% CI = 0.02–1.18; *p* = 0.015].

In the TRIBE bevacizumab cohort, patients with *ERAP1* rs2287987 any C allele (n = 64) showed shorter PFS compared with T/T carriers (n = 151) (8.8 vs. 10.4 months) in both univariate (HR = 1.44, 95% CI = 1.02–2.02; *p* = 0.037) and multivariate analysis (HR = 1.48, 95% CI = 1.04–2.12; *p* = 0.035); *TAP1* rs1135216 any C (n = 50) showed shorter PFS compared with T/T carriers (n = 165) (HR = 1.55, 95% CI = 1.08–2.23; *p* = 0.017) in univariate analysis; *HLA-E* rs1059510 any T allele (n = 99) showed better OS compared with C/C carriers (n = 116) (31.0 vs. 25.1 months) in univariate analysis (HR = 0.72, 95% CI = 0.53–0.98; *p* = 0.036); *HLA-DRA* rs3177928 A/A carriers (n = 5) showed shorter OS compared with any G allele (n = 210) (14.1 vs. 26.8 months) in both univariate (HR = 4.20, 95% CI = 1.70–10.39; *p* = 0.00073) and multivariate (HR = 3.96, 95% CI = 1.50–10.44; *p* = 0.017) analyses; and *HLA-DQB1* rs1063355 any T allele (n = 127) showed shorter PFS compared with G/G carriers (n = 87) (9.5 vs. 10.4 months) in univariate analysis (HR = 1.42, 95% CI = 1.04–1.95; *p* = 0.029).

In the MAVERICC FOLFIRI/bevacizumab cohort, mCRC patients with *ERAP1* rs26653 any C allele (n = 91) showed better PFS compared with G/G carriers (n = 70) (14.5 vs. 10.1 months) in both univariate (HR = 0.57, 95% CI = 0.39–0.83; *p* = 0.0033) and multivariate (HR = 0.54, 95% CI = 0.35–0.84; *p* = 0.0062) analyses; *TAP2* rs241447 C/C carriers (n = 13) showed shorter OS compared with any T allele (n = 150) in univariate analysis (15.5 vs. 27.9 months, HR = 2.40, 95% CI = 1.13–5.09; *p* = 0.018); and *HLA-DMB* rs1042337 G/G carriers (n = 8) showed shorter PFS compared with any A allele (n = 155) (6.1 vs. 12.9 months) in both univariate (HR = 3.77, 95% CI = 1.70–8.33; *p* = 0.00045) and multivariate (HR = 3.33, 95% CI = 1.48–7.48; *p* = 0.011) analyses.

For the FOLFIRI/bevacizumab cohorts in all three trials, no SNPs were found to have significant associations after multiple hypothesis testing corrections.

### 2.4. Association of MHC Class I and II SNPs with Clinical Outcomes in FOLFOX6/Bevacizumab Arm of MAVERICC Trial

**Figure 5:**
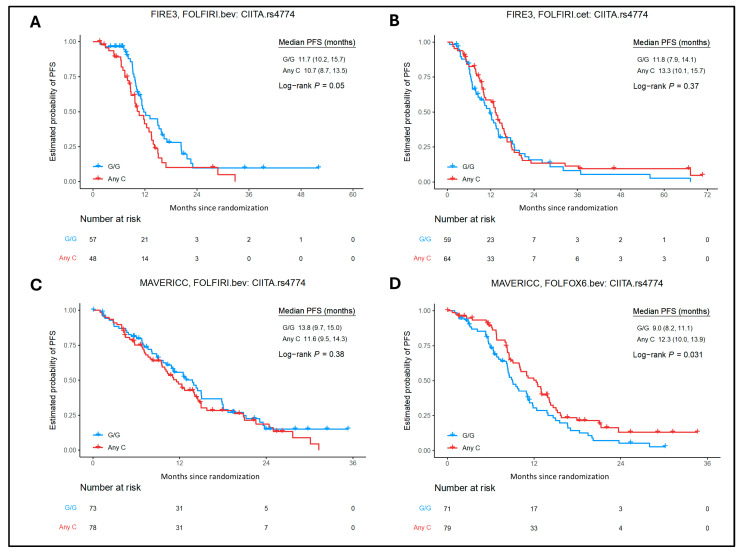
Association between *CIITA* rs4774 expression and patient outcomes in the FIRE-3 and MAVERICC trials. Kaplan–Meier curves show PFS stratified by *CIITA* rs4774 alleles G/G and any C tumor expression according to different treatments of the FIRE-3 and MAVERICC trials. (**A**) FIRE-3 bev/FOLFIRI PFS, (**B**) FIRE-3 cet/FOLFIRI PFS, (**C**) MAVERICC bev/FOLFIRI PFS, (**D**) MAVERICC bev/FOLFOX6 PFS. Bev, bevacizumab; cet, cetuximab; PFS, progression-free survival.

For MHC class I SNPs in the FOLFOX6/bevacizumab arm of the MAVERICC trial, in univariate analysis, *TAP2* rs1800454 any T allele (n = 53) showed better PFS (12.9 vs. 8.8 months, HR = 0.42; 95% CI = 0.19–0.93; *p* = 0.027) and OS (28.7 vs. 22.2 months, HR = 0.58; 95% CI = 0.35–0.97; *p* = 0.036); *TAP2* rs1044043 any A allele (n = 52) showed better OS compared with C/C (n = 109) carriers (28.7 vs. 22.5 months, HR = 0.59; 95% CI = 0.36–0.96; *p* = 0.031); and *HLA-B* rs2769 any A (n = 32) allele showed shorter OS compared with G/G (n = 126) carriers (19.4 vs. 25.9 months, HR = 1.79; 95% CI = 1.06–3.01; *p* = 0.026). For *TAP2* rs1800454, the association with PFS (any T vs. C/C, HR = 0.59; 95% CI = 0.36–0.96; *p* = 0.031) and for *TAP2* rs1044043 the association with OS (any A vs. C/C, HR = 0.59; 95% CI = 0.36–0.96; *p* = 0.031) was confirmed in multivariate analysis. For MHC class II SNPs, in the FOLFOX6/bevacizumab arm of the MAVERICC trial, in univariate analysis *CIITA* rs4774 any C allele (n = 79) showed better PFS compared with G/G (n = 71) carriers (12.3 vs. 11.1 months, HR = 0.67; 95% CI = 0.46–0.97; *p* = 0.031), and was significant in multivariate analysis (*p* = 0.0046) as well as adjusted *p* value (*q* value) < 0.1 (Figure 5). HLA-DPA1 rs1042190 any C allele (n = 71) showed better PFS compared with T/T (n = 90) carriers (11.6 vs. 8.8 months, HR = 0.58; 95% CI = 0.39–0.86; *p* = 0.0063) in multivariate analysis and adjusted *p* value (*q* value) of <0.1 (Tables S4 and S5).

### 2.5. Treatment-by-SNP Interaction

In the dominant genetic model, a significant interaction with treatment was found for *HLA-G* rs1610696 (PFS in FIRE-3), *CIITA* rs4774 (OS in FIRE-3), *ERAP1* rs26653 and *CIITA* rs4774 (PFS in MAVERICC), and *TAP2* rs1044043 (OS in MAVERICC) (Table S5). *HLA-G* rs1610696 showed treatment interaction with targeted agents, with better PFS in the cetuximab-treated cohort. *CIITA* rs4774 showed treatment interaction with both targeted agents and the chemotherapy backbone in FIRE-3 and MAVERICC, respectively. *ERAP1* rs26653 and TAP2 rs1044043 showed interaction with the chemotherapy backbone.

In the recessive genetic model, a significant interaction with treatment was seen for *TAP2* rs241447 and *HLA-DPA1* rs1042190 (PFS in FIRE-3), *TAP2* rs1044043 and *HLA-DRA* rs7192 (OS in FIRE-3) for targeted agents, while *CIITA* rs6498124 and *HLA-DMB* rs1042337 (PFS in MAVERICC) showed interaction with the chemotherapy backbone (Table S6).

### 2.6. Associations Between Selected SNPs and Gene Expression Level in Colon Tissue from GTEx Analysis

The GTEx analysis was obtained for 24 of the total 40 SNPs (significant SNPs selected) tested in the present study. Among MHC class I genes, we found nine SNPs significantly affecting the gene expression status (*p* < 0.05) in normal colon tissue (sigmoid and/or transverse colon): *ERAP1* rs2287987, *ERAP1* rs26653, *TAP2* rs241447, *TAP2* rs1044043, *TAPBP* rs3106191, *HLA-B* rs2769, *HLA-E* rs1059510, and *HLA-G* rs1063320 (Supplementary Figures S1 and S2). Among MHC class II genes, we found five SNPs significantly affecting the gene expression status (*p* < 0.05) in normal colon tissue (sigmoid and/or transverse colon): *CIITA* rs4774, *HLA-DOA* rs3129303, *HLA-DPA1* rs1042190, *HLA-DPB1* rs3097671, *HLA-DQB1* rs1063355, and *HLA-DRA* rs3177928 (Supplementary Figures S3 and S4).

### 2.7. Impact of Selected SNPs on Protein Function and Stability from SIFT and DUET Analysis

**Figure 6:**
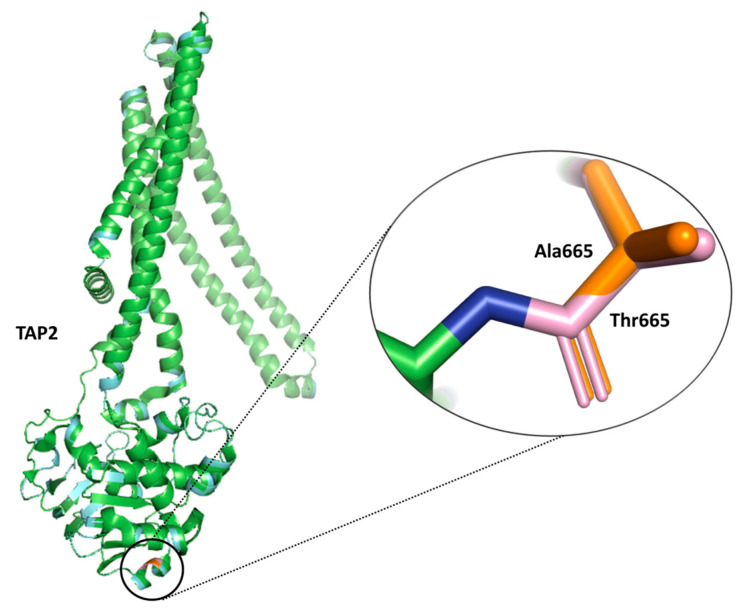
Amino acid substitution of threonine (Thr) to alanine (Ala) at residue number 665 by TAP2 variant rs241447. TAP2 protein structure downloaded from Protein Data Bank (PDB code: 8T46) and visualized in PyMol. Color representation for Thr665 is pink and for Ala665 is orange.

The functional impact on the protein by the SNPs associated with the selected genes from the antigen processing and presentation pathway was assessed using the SIFT server. Out of the 40 SNPs, one SNP rs241447 in *TAP2* showed a deleterious effect on the protein function. The rs241447 variant leads to amino acid substitution from threonine to alanine at position 665. The impact of the T665A mutation on TAP2 protein stability was assessed using DUET and DynaMut servers. Both servers predicted a destabilizing effect of the mutation on the TAP2 protein structure (Figure 6).

## 3. Discussion

Antigen processing and presentation play important roles in adaptive immune response and are mediated by a series of coordinated steps involving multiple proteins. Owing to their important function in normal cells, it has been reported that dysregulation or genetic polymorphisms in the antigen processing and presentation machinery may have implications in several diseases, including sarcoidosis [21], whooping cough [22], cystic fibrosis [23], and NSCLC [24]. The findings in the present study, to the best of our knowledge, reveal for the first time that genetic variations in the antigen processing and presentation pathways are associated with survival outcomes in patients with mCRC, with SNPs showing prognostic and/or predictive association with targeted drugs (cetuximab/bevacizumab) and first-line chemotherapy (irinotecan/oxaliplatin).

The treatment interaction analysis for certain SNPs such as for *ERAP1* and *CIITA* showed interaction with oxaliplatin, which has been shown to cause immunogenic cell death in colorectal cancer cells [25,26] and upregulate MHC class I via the nuclear factor-kappa B (NF-κB) pathway [27]. Our results further support these findings and provide new insights into the association between germline polymorphisms in MHC genes and oxaliplatin efficacy.

Another major finding is the linkage between MHC class I and II SNPs and bevacizumab. Bevacizumab is a recombinant humanized monoclonal IgG1 antibody which targets VEGF-A. Bevacizumab is primarily known to show anti-angiogenic effects; however, reports suggest that it also shows anti-tumor and immune-modulatory effects. VEGF-A inhibits the differentiation and maturation of dendritic cells into their functional form, where they are capable of presenting the tumor antigen on their surface [28,29]. The reports suggested that although VEGF-A inhibits the antigen presentation function of dendritic cells, it did not alter their phenotypic characteristics or induce apoptosis, nor alter the expression of maturation markers including MHC classes I and II [30]. In triple negative breast cancer (TNBC), a VEGF blockade via bevacizumab was shown to increase MHC class I expression and the maturation of memory T cells [31]. However, how the polymorphisms in MHC genes are associated with bevacizumab outcomes in CRC patients remains unknown. Our study showed polymorphisms in MHC genes that were associated with survival outcomes in bevacizumab-treated CRC patients in FIRE-3, TRIBE, and MAVERICC trials, and implicated future studies that could decipher the association between MHC variants and the VEGF-A pathway.

The antigen processing and presentation machinery (APM) play a crucial role in aiding the immune system to recognize and target cancer cells [32]. This particularly raises the potential of utilizing the APM as a therapeutic target for anti-cancer treatment. One such emerging target is ERAP1. ERAPs are responsible for the trimming of antigenic peptides into a size which is suitable for their loading onto HLA (or MHC) class I molecules. The pharmacological inhibition of ERAP1 in the CT26 CRC mouse model using ERAAP (ERAP1 homolog in mice) inhibitor leucinthiol and ERAAP siRNA correlated with tumor regression [33,34]. Greywolf Therapeutics demonstrated their first-in-class ERAP1 inhibitors in combination with anti-PD1 inhibited tumor growth in syngeneic mice tumor models [35]. A previous study reported that any G allele in *ERAP1* rs26654 allele might be associated with a lower risk of cervical cancer compared with C/C carriers (*p* = 0.001) [36]. Additionally, rs26653 is a missense variant and results in P127R amino acid substitution in the ERAP1 protein, which might also have implications on *ERAP1* expression and enzymatic activity [37]. Our GTEx analysis revealed that rs26653 variant alleles significantly affect expression of *ERAP1* in both the sigmoid and transverse colon, with C/C allele increasing *ERAP1* expression. In our analysis, rs26653 C/C carriers had better PFS compared with G/G allele, which might be due to changes in *ERAP1* expression, although how the C/C allele affects *ERAP1* function in CRC tumors remains unknown. Thus, more functional studies are required to determine the effects of these variants on *ERAP1* expression in CRC tumors and their underlying mechanisms.

Epidermal growth factor receptor (EGFR) and vascular endothelial growth factor (VEGF) signaling are the key oncogenic drivers in CRC. EGFR inhibition using cetuximab (monoclonal ligand-blocking antibody) was found to elevate the expression of MHC class I and II molecules in malignant human keratinocytes [38]. Though MHC class I and II gene expression are reportedly linked to the EGFR and VEGF signaling pathways, the clinical implications of genetic alterations in these molecules in mCRC remains largely elusive and unknown.

Apart from the HLA genes which have been extensively studied, other regulatory components of the APM such as those involved in transcriptional regulation (e.g., CIITA) or transport of peptides (e.g., TAP1 and TAP2) are interesting candidates for exploration of their effect on CRC. The antigen peptides, after being processed by the proteasome, enter the endoplasmic reticulum via the heterodimer transporter complex comprising two subunits, TAP1 and TAP2 [39]. *TAP* expression has been found to be dysregulated in several cancers, including breast and melanoma, and mutations in the *TAP* genes in small-cell lung cancer have been implicated in the escape from immune recognition in tumor cells [40]. *TAP2* rs241447 is a missense variant that leads to the amino acid substitution from threonine to alanine at residue position 665 (T665A) [41]. These structural modifications may lead to the disruption of the peptide transport and thus downstream processing of antigenic peptides. *TAP2* rs241447 variants has been recently reported to affect mRNA expression in bladder cancer tissue [42]. GTEx analysis reveals that *TAP2* variants rs241447 and rs1044043 are associated with a change in the expression of TAP2 in the sigmoid and/or transverse colon. A reduction in *TAP2* expression results in the reduced stability and expression of MHC molecules on the tumor cell surface, in turn leading to immune evasion by tumor cells.

Work by Pollack et al. (2011 and 2012) has demonstrated an interesting mechanism by which EGFR inhibitors could influence adaptive immune response by augmenting MHC class I and II gene expression. It has been reported that the transactivation of EGFR along with IFN-γ leads to the attenuation of CIITA induction, and at the same time EGFR inhibitors lead to an augmentation in the induction of CIITA via IFN-γ [38,43]. *CIITA* is a transcriptional activator which coordinates the expression of the entire MHC class II gene family. Controlled and forced expression of *CIITA* in MHC class II-negative hepatocellular cell lines rendered them MHC class II-positive and allowed for better recognition of tumor antigens by CD4+ helper T cells. A combination of existing immunotherapeutic approaches, such as immune-checkpoint inhibitors or oncolytic viruses, with MHC-II expression transcriptionally enhanced by CIITA in cancer cells could potentially increase the recognition of tumor antigen and effector T-cell activation [44]. Treatment interaction in the FIRE-3 cohort showed a significant interaction between CIITA rs4774 and the targeted agents, and based on our results we could hypothesize better OS for mCRC patients with rs4774 C/C variants who were treated with cetuximab compared with bevacizumab. Some studies hint towards the impact of EGFR inhibition on the induction of CIITA expression in non-small cell lung cancer [45]; however, studies are warranted that will validate these findings in CRC and to exploit them for better patient treatment.

Our findings suggest a novel approach of clinical decision making based on the genotype of SNPs in the antigen processing and presentation machinery. Using SNPs MHC I and MCH II may identify patients who would benefit most from combinations including FOLFIRI-bevacizumab, FOLFIRI-cetuximab or FOLFOX6-bevacizumab. An improved understanding of the antigen processing and presentation pathway may allow the development of more effective and less toxic therapies. This study has several limitations. Firstly, this study was performed in a retrospective setting which may introduce selection bias, thus, its prospective validation in clinical trials is warranted/required. Secondly, the prognostic/predictive value of MHC class I and II polymorphisms needs validation in independent cohorts and in vitro/in vivo studies. Thirdly, some statistical limitations need to be mentioned. In certain subgroups, the survival data might appear underpowered and lead to skewed *p*-values and limited sample size, and this prevented us from including some important molecular characteristics such as MSI that are not well represented in the included studies. Lastly, the GTEx analysis provides expression in normal colon tissue and not tumor tissue, and thus the effect of the allele variants on gene expression in the tumor tissue remains unclear. In conclusion, personalized treatment strategy in the choice of targeted drug or backbone chemotherapy based on the genotype of the SNPs reported in the present analysis need to be further validated in future clinical studies.

## 4. Materials and Methods

### 4.1. Patient Samples/Cohorts and Study Design

In the present study, the subjects included were patients with mCRC enrolled in three independent randomized clinical trials, FIRE-3 (NCT00433927) [17], TRIBE (NCT00719797) [18], and MAVERICC [19]. FIRE-3 and TRIBE are Phase III and MAVERICC is a Phase II trial. Patients without sufficient peripheral whole blood samples, SNP data, and/or any other relevant data for analyses were excluded from the study. The FIRE-3 trials included patients randomized to treatment with FOLFIRI plus cetuximab or FOLFIRI plus bevacizumab, the TRIBE trial included patients randomized to treatment with FOLFOXIRI plus bevacizumab or FOLFIRI plus bevacizumab, while the MAVERICC trial included patients with FOLFIRI plus bevacizumab or FOLFOX6 plus bevacizumab. To assess the impact of SNPs associated with MHC class I and II pathway genes on the treatment efficacy, we set the cohorts as follows: cetuximab cohort (FOLFIRI-cetuximab arm of FIRE-3 trial); bevacizumab cohort 1 (FOLFIRI-bevacizumab arm of FIRE-3 trial); bevacizumab cohort 2 (FOLFIRI-bevacizumab arm of TRIBE trial); bevacizumab cohort 3 (FOLFIRI-bevacizumab arm of MAVERICC trial); and bevacizumab cohort 4 (FOLFOX-bevacizumab arm of MAVERICC trial).

### 4.2. Selection of Polymorphisms and Genotyping

Genomic DNA was isolated from the peripheral whole blood samples collected prior to treatment initiation using the QIAamp DNA Blood Mini Kit (Qiagen, Inc., Valencia, CA, USA) in accordance with the manufacturer’s protocol (www.qiagen.com) in the year 2015. Genotyping was performed using the OncoArray platform from Illumina (Illumina, San Diego, CA, USA) containing 530K SNP markers as previously described in Arai et al. (2020) [46]. Ungenotyped SNPs were imputed against the 1000 Genomes Project Phase 3 panel. We focused on 22 genes encoding members of MHC class I and II pathways (*ERAP1*, *ERAP2*, *TAP1*, *TAP2*, *TAPBP*, *β2M*, *HLA-A*, *HLA-B*, *HLA-C*, *HLA-E*, *HLA-F*, *HLA-G*, *CIITA*, *HLA-DMA*, *HLA-DMB*, *HLA-DOA*, *HLA-DOB*, *HLA-DPA1*, *HLA-DPB1*, *HLA-DQA1*, *HLA-DQB1*, and *HLA-DRA*). The candidate SNPs for this study were selected from dbSNP variants (http://www.ncbi.nlm.nih.gov) based on fulfilling the following criteria: (1) minor allele frequency in Caucasians (defined as ‘European’ in 1000 Genomes Project phase III) of equal to or greater than 10% based on the Ensembl Genome Browser (https://www.ensembl.org); (2) having potential biological functions, including transcription factor-binding site, splicing site, miRNA-binding site, nonsynonymous coding, stop codon, and drug sensitivity, based on the published literature and/or public databases such as https://snpinfo.niehs.nih.gov and https://www.ncbi.nlm.nih.gov; (3) tag SNPs with an r^2^ threshold of 0.8 selected from HapMap genotype data (https://snpinfo.niehs.nih.gov). Using the LDlink suite (https://ldlink.nci.nih.gov), SNPs showing a linkage disequilibrium (LD) of R^2^ > 0.8 were excluded from the study. The above-mentioned databases were accessed from October-November 2022 for the present study. In total, 40 SNPs met the inclusion criteria for this study and were used for further analysis: *ERAP1* (rs30187, rs26653, rs2287987, rs13160562), *ERAP2* (rs41506651, rs2549782), *TAP1* (rs1135216), *TAP2* (rs241447, rs1800454, rs1044043), *TAPBP* (rs2071888, rs3106191), *β2M* (rs2255235), *HLA-A* (rs2499, rs2770), *HLA-B* (rs1058026, rs2769, rs1049853), *HLA-C* (rs35075694, rs1049281), *HLA-E* (rs1059510), *HLA-F* (rs1736924), *HLA-G* (rs1610696, rs1063320), *CIITA* (rs4774, rs1139564, rs6498124), *HLA-DMA* (rs1063478), *HLA-DMB* (rs1042337, rs10751), *HLA-DOA* (rs375256, rs9276975, rs3129303), *HLA-DOB* (rs11244), *HLA-DPA1* (rs1042190), *HLA-DPB1* (rs3097671), *HLA-DQA1* (rs707952), *HLA-DQB1* (rs1063355), and *HLA-DRA* (rs7192, rs3177928). The characteristics of the selected SNPs are represented in Tables S1 and S2.

### 4.3. Statistical Analysis

The 40 selected SNPs, associated with the 22 genes involved in MHC class I and II pathways, were evaluated for possible associations with tumor response, PFS, and OS based on the three genetic models (additive, dominant, and recessive). PFS was defined as the time from randomization to disease progression or death from any cause. OS was defined as the time from randomization to death from any cause. Patients who did not experience any events were censored at the last follow-up date. Log-rank tests were used to evaluate associations between SNPs and PFS or OS in univariate analyses and likelihood ratio tests were used for multivariable analyses with Cox proportional hazards models. Trial-specific adjustment covariates for FIRE-3 were age, sex, Eastern Cooperative Oncology Group (ECOG), performance status (PS), liver-limited disease, RAS/BRAF status, and tumor sidedness; for TRIBE, they were age, sex, ECOG PS, liver-limited disease, RAS/BRAF status, tumor sidedness, adjuvant chemotherapy, and primary tumor resection; and for MAVERICC, they were age, sex, ECOG, RAS status, ethnicity, primary tumor resection, number of metastatic sites, and tumor sidedness. The first three principal components (PCs) computed from ancestry informative markers (AIMs) included on the OncoArray were included as additional adjustment covariates. *p* values were adjusted for multiple comparisons by computing the False Discovery Rate (FDR) and represented as *q* values. Benjamini and Hochberg FDR (BH FDR) was computed separately for each study arm, each outcome, and MHC I vs. MHC II pathways. FDR was computed solely for multivariable likelihood ratio tests, grouping additive, dominant, and recessive models [47]. To formally assess the predictive value of SNPs, treatment-by-SNP interactions were assessed in multivariable Cox models as described above by including an additional product term with the SNP coded additively and a binary indicator for treatment (single degree-of-freedom). The corresponding interaction parameter estimates were evaluated and *p*-values generated using likelihood ratio tests.

The online server Genotype-Tissue Expression (GTEx) portal v8 (https://www.gtexportal.org/home/) was accessed on 24 February 2025 to assess the effect of the selected SNPs on gene expression in normal colon tissue (sigmoid and transverse colon). The Sort Intolerant from Tolerant (SIFT) algorithm (https://sift.bii.a-star.edu.sg/) was accessed on June-July 2024 to assess the functional impact of the SNP and associated amino acid substitution on the protein function, and DUET (https://biosig.lab.uq.edu.au/duet/stability) and DynaMut (https://biosig.lab.uq.edu.au/dynamut2/) online tools were accessed on June-July 2024 to assess the impact of the selected SNPs on the protein stability.

## 5. Conclusions

In conclusion, our results provide the first evidence for an important role for MHC SNPs as prognostic and predictive biomarkers for first-line treatment in mCRC, with differential effects based on the biologic agent (cetuximab/bevacizumab) and chemotherapy backbone (irinotecan/oxaliplatin). These biomarkers, when further validated, may contribute to personalized treatment approaches for mCRC patients. These findings may also provide insight into the potential efficacy of emerging ERAP1 inhibitors when combined with targeted drug and immunotherapeutic treatment in mCRC patients. The novel findings reported in our study warrant further preclinical and prospective clinical studies for validation.

## Data Availability

Data are available upon reasonable request. The study protocol and statistical analysis plan are available in the paper. Other data (including the summary of clinical and genomic data) will be made available upon reasonable request. (pm_344@usc.edu, fbattagl@usc.edu, lenz@usc.edu).

## Supplementary Materials

The following supporting information can be downloaded at: https://www.mdpi.com/article/10.3390/ijms26062556/s1.

## Abbreviations

The following abbreviations are used in this manuscript:

AIM	Ancestry informative markers
APC	Antigen presenting cells
APM	Antigen processing and presentation machinery
B2M	Beta-2-microglobulin
CRC	Colorectal Cancer
ECOG	Eastern Cooperative Oncology Group
EGFR	Epidermal growth factor receptor
ERAP1/2	Endoplasmic reticulum aminopeptidases ½
GTEx	Genotype-Tissue Expression
HLA	Human Leukocyte Antigens
KIRs	Killer cell immunoglobulin-like receptors
LD	Linkage disequilibrium
MHC	Major Histocompatibility Complex
NF-κB	Nuclear factor-kappa B
NK	Natural killer cells
NSCLC	Non-small cell lung cancer
OS	Overall Survival
PC	Principal components
PFS	Progression free survival
PS	Performance status
SIFT	Sort Intolerant from Tolerant
SNP	Single nucleotide polymorphisms
TAP1/2	Transporter associated with antigen processing 1/2
TAPBP	Tapasin/TAP-binding protein
TNBC	Triple negative breast cancer
VEGF	Vascular endothelial growth factor
