# Supplementary material for: Genetic Polymorphisms in MHC Classes I and II Predict Outcomes in Metastatic Colorectal Cancer
Source: Int J Mol Sci. 2025 Mar 12;26(6):2556. doi: 10.3390/ijms26062556 (PMC11942614; doi:10.3390/ijms26062556)
Supplement: Supplementary file 1 [file ijms-26-02556-s001.zip › ijms-3475627-supplementary.docx]

**Supplementary File**

**Table S1 Characteristics of selected SNPs in MHC class I pathway.**

| **Gene name** | **SNP** | **Chromosome** | **Base Exchange** | **Function/ location** | **MAF (CEU)** |
| --- | --- | --- | --- | --- | --- |
| **ERAP1** | **rs30187** | 5 | T>C | missense variant | 0.313 |
|  | **rs26653** | 5 | G>C | missense variant | 0.278 |
|  | **rs2287987** | 5 | T>C | missense variant | 0.263 |
|  | **rs13160562** | 5 | G>A | 3 prime UTR variant | 0.364 |
| **ERAP2** | **rs41506651** | 5 | C>T/C>G | missense variant | 0.116 |
|  | **rs2549782** | 5 | T>G/T>C | missense variant | 0.5 |
| **TAP1** | **rs1135216** | 6 | C>T | missense variant | 0.111 |
| **TAP2** | **rs241447** | 6 | T>C/T>G | missense variant | 0.268 |
|  | **rs1800454** | 6 | C>T | missense variant | 0.167 |
|  | **rs1044043** | 6 | C>A/C>G | 3 prime UTR variant | 0.232 |
| **TAPBP** | **rs2071888** | 6 | C>G | missense variant | 0.465 |
|  | **rs3106191** | 6 | C>A | 5 prime UTR variant | 0.263 |
| **B2M** | **rs2255235** | 15 | T>G/A/C | 5 prime UTR variant | 0.126 |
| **HLA-A** | **rs2499** | 6 | G>T/C/A | 3 prime UTR variant | 0.141 |
| **HLA-B** | **rs2770** | 6 | A>G | 3 prime UTR variant | 0.48 |
|  | **rs1058026** | 6 | A>C | 3 prime UTR variant | 0.121 |
|  | **rs2769** | 6 | G>A | 3 prime UTR variant | 0.202 |
| **HLA-C** | **rs1049853** | 6 | G>A/C | 3 prime UTR variant | 0.136 |
|  | **rs35075694** | 6 | G>A | 3 prime UTR variant | 0.146 |
|  | **rs1049281** | 6 | T>C/G | 3 prime UTR variant | 0.348 |
| **HLA-E** | **rs1059510** | 6 | C>T/G/A | missense variant | 0.354 |
| **HLA-F** | **rs1736924** | 6 | T>C/A/G | missense variant | 0.192 |
| **HLA-G** | **rs1610696** | 6 | C>G | 3 prime UTR variant | 0.253 |
|  | **rs1063320** | 6 | G>C/T | 3 prime UTR variant | 0.44 |

**Table S2 Characteristics of selected SNPs in MHC class II pathway.**

| **Gene name** | **SNP** | **Chromosome** | **Base Exchange** | **Function/ location** | **MAF (CEU)** |
| --- | --- | --- | --- | --- | --- |
| **CIITA** | **rs4774** | 16 | C>G | missense variant | 0.283 |
|  | **rs1139564** | 16 | T>C | 3 prime UTR variant | 0.141 |
|  | **rs6498124** | 16 | G>T/G>A | 3 prime UTR variant | 0.444 |
| **HLA-DMA** | **rs1063478** | 6 | C>T | missense variant | 0.121 |
| **HLA-DMB** | **rs1042337** | 6 | A>G/A>C | missense variant | 0.268 |
|  | **rs10751** | 6 | G>A | 3 prime UTR variant | 0.167 |
| **HLA-DOA** | **rs375256** | 6 | G>A/G>C | missense variant | 0.247 |
|  | **rs9276975** | 6 | C>T | 3 prime UTR variant | 0.162 |
|  | **rs3129303** | 6 | T>C | 3 prime UTR variant | 0.162 |
| **HLA-DOB** | **rs11244** | 6 | G>A | 3 prime UTR variant | 0.232 |
| **HLA-DPA1** | **rs1042190** | 6 | T>C/T>G | missense variant | 0.182 |
| **HLA-DPB1** | **rs3097671** | 6 | G>C | non coding transcript exon variant | 0.126 |
| **HLA-DQA1** | **rs707952** | 6 | C>T/C>A | missense variant | 0.146 |
| **HLA-DQB1** | **rs1063355** | 6 | T>C/T>G | 3 prime UTR variant | 0.404 |
| **HLA-DRA** | **rs7192** | 6 | G>T/G>C | missense variant | 0.333 |
|  | **rs3177928** | 6 | G>A | 3 prime UTR variant | 0.182 |

**Table S3 Associations between SNPs associated with *MHC class I* genes and clinical outcomes.**

| **SNP** | **Genotype** | **N** |  | **PFS** | | | | | | **OS** | | | | |
| --- | --- | --- | --- | --- | --- | --- | --- | --- | --- | --- | --- | --- | --- | --- |
|  |  |  |  |  | **Median PFS (months)** | **Univariate Analysis** | | **Multivariate Analysis** | | **Median OS (months)** | **Univariate Analysis** | | **Multivariate Analysis** | |
|  |  |  |  |  |  | **HR (95% CI)** | **P** | **HR (95% CI)** | **P** |  | **HR (95% CI)** | **P** | **HR (95% CI)** | **P** |
| **FIRE-3** FOLFIRI-**Cetuximab Cohort** | | | | | | | | | | | | | | |
| TAP1  rs1135216 | T/T | 96 |  |  | 12.2 (9.5, 14.0) | 1 | 0.69 | 1 | 0.91 | 42.0 (36.3, 60.6) | 1 | **0.042** | 1 | 0.11 |
|  | Any C | 33 |  |  | 13.3 (10.3, 17.7) | 0.92 (0.58, 1.43) |  | 1.03 (0.64, 1.64) |  | 58.9 (49.7, --) | 0.43 (0.19, 0.99) |  | 0.52 (0.22, 1.22) |  |
| TAP2  rs241447 | Any T | 119 |  |  | 12.8 (10.2, 14.1) | 1 | 0.28 | 1 | 0.27 | 51.9 (42.0, 67.2) | 1 | **0.023** | 1 | **0.035** |
|  | C/C | 10 |  |  | 10.1 (1.4, --) | 1.53 (0.70, 3.34) |  | 1.60 (0.73, 3.50) |  | 28.6 (9.0, --) | 2.91 (1.11, 7.67) |  | 3.35 (1.24, 9.08) |  |
| TAP2 rs1800454 | C/C | 93 |  |  | 12.2 (9.0, 14.1) | 1 | 0.69 | 1 | 0.43 | 42.7 (29.9, 60.6) | 1 | **0.042** | 1 | 0.16 |
|  | Any T | 36 |  |  | 12.8 (10.1, 15.8) | 0.92 (0.60, 1.41) |  | 1.20 (0.76, 1.90) |  | 58.9 (40.8, --) | 0.42 (0.17, 0.99) |  | 0.54 (0.22, 1.34) |  |
| TAP2 rs1044043 | C/C | 77 |  |  | 12.2 (9.3, 13.5) | 1 | 0.16 | 1 | **0.021** | 56.1 (40.8, --) | 1 | 0.58 | 1 | 0.86 |
|  | Any A | 52 |  |  | 13.9 (9.9, 16.5) | 0.76 (0.51, 1.13) |  | 0.62 (0.40, 0.94) |  | 42.0 (27.1, 67.2) | 1.19 (0.64, 2.22) |  | 0.94 (0.48, 1.84) |  |
| TAP2 rs1044043 | Any C | 119 |  |  | 12.1 (9.9, 13.5) | 1 | **0.045** | 1 | **0.0038** | 51.9 (40.8, 60.6) | 1 | 0.77 | 1 | 0.71 |
|  | A/A | 10 |  |  | 32.1 (3.6, 36.9) | 0.47 (0.22, 1.02) |  | 0.34 (0.15, 0.78) |  | 42.7 (16.1, --) | 1.17 (0.41, 3.29) |  | 0.81 (0.26, 2.53) |  |
| TAPBP rs3106191 | C/C | 63 |  |  | 15.1 (10.6, 17.9) | 1 | **0.0049** | 1 | **0.0026** | 58.9 (40.0, --) | 1 | 0.52 | 1 | 0.33 |
|  | Any A | 66 |  |  | 10.3 (8.1, 12.8) | 1.75 (1.18, 2.59) |  | 1.86 (1.24, 2.79) |  | 49.7 (28.6, --) | 1.23 (0.66, 2.28) |  | 1.37 (0.72, 2.59) |  |
| HLA-B rs2770 | A/A | 20 |  |  | 6.5 (5.2, 18.5) | 1 | 0.13 | 1 | 0.36 | 37.4 (14.1, --) | 1 | **0.027** | 1 | **0.027** |
|  | Any G | 67 |  |  | 12.2 (10.1, 14.1) | 0.65 (0.37, 1.15) |  | 0.74 (0.40, 1.38) |  | 56.1 (42.7, --) | 0.42 (0.19, 0.93) |  | 0.38 (0.16, 0.86) |  |
| HLA-G rs1610696 | C/C | 50 |  |  | 10.8 (7.9, 13.3) | 1 | **0.046** | 1 | 0.077 | 42.0 (20.5, --) | 1 | 0.15 | 1 | 0.18 |
|  | Any G | 77 |  |  | 14.0 (10.6, 16.5) | 0.67 (0.45, 1.00) |  | 0.69 (0.46, 1.04) |  | 51.9 (40.0, 67.2) | 0.64 (0.34, 1.19) |  | 0.64 (0.33, 1.22) |  |
| **FIRE-3** FOLFIRI-**Bevacizumab Cohort** | | | | | | | | | | | | | | |
| ERAP1 rs2287987 | Any T | 102 |  |  | 11.7 (10.2, 13.5) | 1 | **0.04** | 1 | **0.049** | 31.4 (25.0, 48.3) | 1 | 0.57 | 1 | 0.33 |
|  | C/C | 5 |  |  | 9.9 (3.7, --) | 2.81 (1.00, 7.89) |  | 3.46 (1.18, 10.12) |  | 28.6 (11.5, --) | 1.41 (0.43, 4.57) |  | 1.94 (0.56, 6.71) |  |
| **TRIBE** FOLFIRI-**Bevacizumab Cohort** | | | | | | | | | | | | | | |
| ERAP1 rs2287987 | T/T | 151 |  |  | 10.4 (9.4, 11.5) | 1 | **0.037** | 1 | **0.035** | 26.9 (22.7, 31.6) | 1 | 0.98 | 1 | 0.68 |
|  | Any C | 64 |  |  | 8.8 (7.8, 10.6) | 1.44 (1.02, 2.02) |  | 1.48 (1.04, 2.12) |  | 25.9 (18.8, 37.6) | 1.00 (0.71, 1.39) |  | 1.08 (0.76, 1.52) |  |
| TAP1 rs1135216 | T/T | 165 |  |  | 10.8 (9.5, 11.5) | 1 | **0.017** | 1 | 0.055 | 26.5 (22.4, 31.6) | 1 | 0.99 | 1 | 0.62 |
|  | Any C | 50 |  |  | 8.8 (7.9, 10.3) | 1.55 (1.08, 2.23) |  | 1.46 (1.00, 2.13) |  | 26.2 (18.3, 37.2) | 1.00 (0.70, 1.44) |  | 0.91 (0.62, 1.33) |  |
| HLA-E rs1059510 | C/C | 116 |  |  | 9.5 (8.7, 10.5) | 1 | 0.32 | 1 | 0.43 | 25.1 (20.1, 29.8) | 1 | **0.036** | 1 | 0.096 |
|  | Any T | 99 |  |  | 11.1 (9.3, 12.2) | 0.85 (0.63, 1.16) |  | 0.88 (0.64, 1.21) |  | 31.0 (25.0, 38.4) | 0.72 (0.53, 0.98) |  | 0.76 (0.55, 1.05) |  |
| MAVERICC FOLFIRI-**Bevacizumab Cohort** | | | | | | | | | | | | | | |
| ERAP1 rs26653 | G/G | 70 |  |  | 10.1 (7.1, 12.6) | 1 | **0.0033** | 1 | **0.0062** | 24.6 (17.0, --) | 1 | 0.079 | 1 | 0.16 |
|  | Any C | 91 |  |  | 14.5 (12.3, 18.0) | 0.57 (0.39, 0.83) |  | 0.54 (0.35, 0.84) |  | 27.9 (26.9, --) | 0.64 (0.39, 1.06) |  | 0.67 (0.38, 1.17) |  |
| TAP2 rs241447 | Any T | 150 |  |  | 12.6 (10.3, 14.5) | 1 | 0.97 | 1 | 0.95 | 27.9 (26.5, --) | 1 | **0.018** | 1 | **0.019** |
|  | C/C | 13 |  |  | 11.0 (4.9, --) | 1.01 (0.47, 2.20) |  | 0.98 (0.44, 2.17) |  | 15.5 (12.4, --) | 2.40 (1.13, 5.09) |  | 2.87 (1.30, 6.33) |  |
| MAVERICC FOLFOX-**Bevacizumab Cohort** | | | | | | | | | | | | | | |
| TAP2 rs1800454 | C/C | 108 |  |  | 8.8 (8.3, 11.0) | 1 | **0.013** | 1 | **0.029** | 22.2 (18.7, 26.1) | 1 | **0.036** | 1 | 0.055 |
|  | Any T | 53 |  |  | 12.9 (10.9, 15.5) | 0.61 (0.41, 0.90) |  | 0.64 (0.43, 0.96) |  | 28.7 (22.7, --) | 0.58 (0.35, 0.97) |  | 0.61 (0.36, 1.03) |  |
| TAP2 rs1044043 | C/C | 109 |  |  | 10.9 (8.5, 12.5) | 1 | 0.42 | 1 | 0.51 | 22.5 (18.8, 25.5) | 1 | **0.031** | 1 | **0.04** |
|  | Any A | 52 |  |  | 10.1 (8.5, 12.3) | 1.17 (0.81, 1.69) |  | 1.14 (0.77, 1.67) |  | 28.7 (22.8, 37.4) | 0.59 (0.36, 0.96) |  | 0.59 (0.36, 0.99) |  |
| HLA-B rs2769 | G/G | 126 |  |  | 10.9 (8.6, 12.4) | 1 | 0.62 | 1 | 0.84 | 25.9 (22.8, 30.4) | 1 | **0.026** | 1 | 0.062 |
|  | Any A | 32 |  |  | 10.0 (8.5, 12.5) | 1.12 (0.71, 1.76) |  | 1.05 (0.65, 1.68) |  | 19.4 (16.9, 22.2) | 1.79 (1.06, 3.01) |  | 1.71 (0.99, 2.93) |  |
| HLA-C rs1049281 | Any C | 149 |  |  | 10.2 (8.8, 11.9) | 1 | 0.87 | 1 | 0.51 | 23.2 (20.1, 26.1) | 1 | 0.064 | 1 | **0.019** |
|  | T/T | 12 |  |  | 11.0 (8.0, 14.7) | 0.95 (0.50, 1.82) |  | 0.81 (0.41, 1.57) |  | 37.4 (16.9, --) | 0.40 (0.15, 1.09) |  | 0.34 (0.12, 0.96) |  |
| HLA-G rs1063320 | G/G | 48 |  |  | 11.4 (8.5, 14.7) | 1 | 0.44 | 1 | 0.69 | 23.9 (18.6, 28.7) | 1 | 0.29 | 1 | **0.048** |
|  | Any C | 113 |  |  | 10.0 (8.5, 11.8) | 1.17 (0.79, 1.74) |  | 1.09 (0.72, 1.64) |  | 25.5 (20.1, 30.4) | 0.78 (0.49, 1.23) |  | 0.60 (0.37, 0.98) |  |

Footnote: Although all the SNPs mentioned in the paper were analyzed, only the SNPs showing significant P values (<0.05) were represented in the above table.

Significant P values are shown in bold characters.

Abbreviation: CI, confidence interval; HR, hazard ratio; ORR, overall response rate; OS, overall survival; PFS, progression-free survival; SNP, single-nucleotide polymorphism.

**Table S4 Associations between SNPs associated with *MHC class II* genes and clinical outcomes.**

| **SNP** | **Genotype** | **N** |  | **PFS** | | | | | | **OS** | | | | |
| --- | --- | --- | --- | --- | --- | --- | --- | --- | --- | --- | --- | --- | --- | --- |
|  |  |  |  |  | **Median PFS (months)** | **Univariate Analysis** | | **Multivariate Analysis** | | **Median OS (months)** | **Univariate Analysis** | | **Multivariate Analysis** | |
|  |  |  |  |  |  | **HR (95% CI)** | **P** | **HR (95% CI)** | **P** |  | **HR (95% CI)** | **P** | **HR (95% CI)** | **P** |
| **FIRE-3** FOLFIRI-**Cetuximab Cohort** | | | | | | | | | | | | | | |
| CIITA rs4774 | G/G | 59 |  |  | 11.8 (7.9, 14.1) | 1 | 0.37 | 1 | 0.48 | 42.0 (29.9, 56.1) | 1 | 0.063 | 1 | **0.039** |
|  | Any C | 64 |  |  | 13.3 (10.1, 15.7) | 0.84 (0.56, 1.24) |  | 0.86 (0.58, 1.29) |  | 60.6 (37.4, --) | 0.54 (0.28, 1.04) |  | 0.50 (0.25, 0.98) |  |
| HLA-DMB rs10751 | G/G | 94 |  |  | 12.9 (10.6, 14.5) | 1 | 0.054 | 1 | **0.03** | 49.7 (40.0, 67.2) | 1 | 0.23 | 1 | 0.21 |
|  | Any A | 24 |  |  | 9.5 (6.9, 13.4) | 1.58 (0.99, 2.54) |  | 1.76 (1.08, 2.87) |  | 37.4 (19.1, --) | 1.59 (0.74, 3.41) |  | 1.73 (0.75, 3.99) |  |
| HLA-DOB rs11244 | Any G | 122 |  |  | 12.8 (10.2, 14.1) | 1 | **0.045** | 1 | 0.09 | 49.7 (40.0, 60.6) | 1 | 0.33 | 1 | 0.14 |
|  | A/A | 7 |  |  | 7.8 (1.9, 14.1) | 2.18 (1.00, 4.75) |  | 2.12 (0.96, 4.67) |  | -- (12.9, --) | 0.39 (0.05, 2.84) |  | 0.28 (0.04, 2.15) |  |
| HLA-DPB1 rs3097671 | G/G | 91 |  |  | 13.5 (10.8, 16.1) | 1 | **0.014** | 1 | **0.014** | 49.7 (40.8, 60.6) | 1 | 0.57 | 1 | 0.44 |
|  | Any C | 38 |  |  | 9.9 (6.9, 12.3) | 1.69 (1.11, 2.58) |  | 1.76 (1.14, 2.73) |  | 67.2 (23.9, --) | 1.23 (0.61, 2.47) |  | 1.33 (0.65, 2.73) |  |
| HLA-DRA rs7192 | G/G | 47 |  |  | 8.7 (6.4, 12.2) | 1 | **0.014** | 1 | **0.043** | 40.8 (27.1, 58.9) | 1 | **0.039** | 1 | **0.044** |
|  | Any T | 82 |  |  | 13.9 (12.2, 15.8) | 0.62 (0.42, 0.92) |  | 0.65 (0.44, 0.98) |  | 56.1 (46.4, --) | 0.53 (0.29, 0.98) |  | 0.52 (0.28, 0.98) |  |
| **FIRE-3** FOLFIRI-**Bevacizumab Cohort** | | | | | | | | | | | | | | |
| CIITA rs4774 | Any G | 95 |  |  | 11.4 (9.9, 13.5) | 1 | 0.24 | 1 | 0.9 | 39.9 (25.8, 49.8) | 1 | **0.02** | 1 | 0.34 |
|  | C/C | 10 |  |  | 11.7 (1.9, 14.2) | 1.60 (0.73, 3.53) |  | 1.07 (0.38, 3.05) |  | 20.1 (3.4, --) | 2.55 (1.13, 5.77) |  | 1.57 (0.64, 3.85) |  |
| HLA-DMB rs1042337 | Any A | 102 |  |  | 11.4 (9.9, 12.9) | 1 | 0.064 | 1 | **0.015** | 31.4 (24.8, 48.3) | 1 | 0.32 | 1 | 0.16 |
|  | G/G | 5 |  |  | 21.7 (21.7, --) | 0.19 (0.03, 1.36) |  | 0.16 (0.02, 1.18) |  | 28.8 (28.8, --) | 0.38 (0.05, 2.79) |  | 0.29 (0.04, 2.27) |  |
| **TRIBE** FOLFIRI-**Bevacizumab Cohort** | | | | | | | | | | | | | | |
| HLA-DQB1 rs1063355 | G/G | 87 |  |  | 10.4 (8.8, 12.3) | 1 | **0.029** | 1 | 0.18 | 29.8 (23.1, 37.9) | 1 | 0.054 | 1 | 0.12 |
|  | Any T | 127 |  |  | 9.5 (8.4, 10.8) | 1.42 (1.04, 1.95) |  | 1.26 (0.90, 1.76) |  | 25.5 (20.5, 30.3) | 1.36 (0.99, 1.87) |  | 1.30 (0.93, 1.82) |  |
| HLA-DRA rs3177928 | Any G | 210 |  |  | 9.9 (9.3, 11.0) | 1 | 0.054 | 1 | 0.08 | 26.8 (24.8, 31.6) | 1 | **0.00073** | 1 | **0.017** |
|  | A/A | 5 |  |  | 7.2 (1.9, --) | 2.58 (0.95, 7.00) |  | 2.94 (1.02, 8.46) |  | 14.1 (5.2, --) | 4.20 (1.70, 10.39) |  | 3.96 (1.50, 10.44) |  |
| **MAVERICC** FOLFIRI-**Bevacizumab Cohort** | | | | | | | | | | | | | | |
| HLA-DMB rs1042337 | Any A | 155 |  |  | 12.9 (11.0, 14.8) | 1 | **0.00045** | 1 | **0.011** | 27.5 (25.0, --) | 1 | 0.09 | 1 | 0.23 |
|  | G/G | 8 |  |  | 6.1 (2.3, --) | 3.77 (1.70, 8.33) |  | 3.33 (1.48, 7.48) |  | 18.8 (6.3, --) | 2.17 (0.87, 5.42) |  | 1.87 (0.73, 4.76) |  |
| **MAVERICC** FOLFOX-**Bevacizumab Cohort** | | | | | | | | | | | | | | |
| CIITA rs4774 | G/G | 71 |  |  | 9.0 (8.2, 11.1) | 1 | **0.031** | 1 | **0.0046** | 19.8 (16.9, 24.3) | 1 | 0.059 | 1 | **0.012** |
|  | Any C | 79 |  |  | 12.3 (10.0, 13.9) | 0.67 (0.46, 0.97) |  | 0.57 (0.38, 0.84) |  | 26.1 (22.7, 36.0) | 0.65 (0.42, 1.02) |  | 0.55 (0.34, 0.88) |  |
| CIITA rs1139564 | Any C | 117 |  |  | 10.1 (8.6, 11.9) | 1 | 0.34 | 1 | 0.18 | 24.3 (20.4, 30.4) | 1 | 0.11 | 1 | **0.015** |
|  | T/T | 5 |  |  | 6.4 (3.4, --) | 1.63 (0.59, 4.47) |  | 2.28 (0.76, 6.87) |  | 18.7 (3.4, --) | 2.06 (0.82, 5.15) |  | 4.94 (1.55, 15.74) |  |
| HLA-DOA rs375256 | G/G | 94 |  |  | 11.4 (9.1, 13.0) | 1 | **0.038** | 1 | **0.033** | 26.1 (22.8, 30.4) | 1 | 0.12 | 1 | 0.46 |
|  | Any A | 67 |  |  | 8.5 (8.3, 11.2) | 1.46 (1.02, 2.10) |  | 1.53 (1.04, 2.24) |  | 19.2 (15.7, 24.8) | 1.40 (0.91, 2.16) |  | 1.20 (0.75, 1.91) |  |
| HLA-DOA rs3129303 | T/T | 109 |  |  | 11.0 (9.1, 12.5) | 1 | 0.059 | 1 | **0.038** | 22.8 (18.8, 26.1) | 1 | 0.54 | 1 | 0.86 |
|  | Any C | 50 |  |  | 8.6 (7.1, 12.0) | 1.44 (0.98, 2.11) |  | 1.52 (1.03, 2.24) |  | 27.3 (22.2, 34.0) | 0.86 (0.54, 1.38) |  | 1.04 (0.64, 1.70) |  |
| HLA-DPA1 rs1042190 | T/T | 90 |  |  | 8.8 (8.3, 11.4) | 1 | 0.051 | 1 | **0.0063** | 23.2 (19.2, 30.4) | 1 | 0.96 | 1 | 0.51 |
|  | Any C | 71 |  |  | 11.6 (10.0, 14.3) | 0.70 (0.48, 1.00) |  | 0.58 (0.39, 0.86) |  | 25.5 (20.4, 28.7) | 0.99 (0.64, 1.53) |  | 0.85 (0.53, 1.36) |  |
| HLA-DRA rs7192 | Any G | 139 |  |  | 11.0 (9.0, 12.4) | 1 | 0.12 | 1 | **0.039** | 24.3 (20.4, 27.3) | 1 | 0.85 | 1 | 0.69 |
|  | T/T | 22 |  |  | 8.8 (6.2, 12.3) | 1.50 (0.90, 2.48) |  | 1.82 (1.06, 3.13) |  | 28.7 (13.8, 37.4) | 0.94 (0.51, 1.76) |  | 1.14 (0.60, 2.19) |  |

Footnote: Although all the SNPs mentioned in the paper were analyzed, only the SNPs showing significant P values (<0.05) were represented in the above table.

Significant P values are shown in bold characters.

Abbreviation: CI, confidence interval; HR, hazard ratio; ORR, overall response rate; OS, overall survival; PFS, progression-free survival; SNP, single-nucleotide polymorphism.

**Table S5. Multiple hypothesis testing corrections for MHC class I and II SNPs in FIRE-3, TRIBE and MAVERICC trials.**

| **SNP** | **Genotype** | **N** | **Univariate PFS**  ***P* value** | **Multivariate PFS**  ***P* value** | **Univariate OS**  ***P* value** | **Multivariate OS**  ***P* value** | ***q* value PFS (Adjusted *P* value)** | ***q* value OS (Adjusted *P* value)** | **Study** | **Treatment** |
| --- | --- | --- | --- | --- | --- | --- | --- | --- | --- | --- |
| *TAP2* rs1044043 | Any C | 119 | **0.045** | **0.0038** | 0.77 | 0.71 | **0.087** | 0.914615385 | FIRE-3 | FOLFIRI.cet |
|  | A/A | 10 |  |  |  |  |  |  |  |  |
| *TAPBP* rs3106191 | C/C | 63 | **0.0049** | **0.0026** | 0.52 | 0.33 | **0.087** | 0.754 | FIRE-3 | FOLFIRI.cet |
|  | Any A | 66 |  |  |  |  |  |  |  |  |
| *CIITA* rs4774 | G/G | 71 | **0.031** | **0.0046** | 0.059 | 0.012 | **0.0819** | 0.2925 | MAVERICC | FOLFOX6.bev |
|  | Any C | 79 |  |  |  |  |  |  |  |  |
| *HLA-DPA1* rs1042190 | T/T | 90 | 0.051 | **0.0063** | 0.96 | 0.51 | **0.0819** | 0.82875 | MAVERICC | FOLFOX6.bev |
|  | Any C | 71 |  |  |  |  |  |  |  |  |

**Table S6. Treatment-by-SNP interaction test (only the significant results *P* < 0.05)**

| **Gene (SNP)** | **Dominant Model** | | **Recessive Model** | |
| --- | --- | --- | --- | --- |
|  | **PFS Interaction P** | **OS Interaction P** | **PFS Interaction P** | **OS Interaction P** |
| **FIRE3** | | | | |
| *ERAP1* (rs26653) | **0.039534** | 0.361936 |  |  |
| *HLA-G* (rs1610696) | **0.023688** | **0.030743** |  |  |
| *TAP2* (rs241447) |  |  | 0.702373 | **0.023753** |
| *CIITA* (rs4774) | 0.087879 | **0.013316** |  |  |
| *HLA-DRA* (rs7192) | 0.066181 | 0.073379 | 0.129804 | **0.023856** |
| *HLA-DPA1* (rs1042190) |  |  | **0.006949** | 0.108275 |
| MAVERICC | | | | |
| *ERAP1* (rs26653) | **0.002288** | 0.227082 |  |  |
| *TAP2* (rs1044043) | 0.858883 | **0.031753** |  |  |
| *CIITA* (rs4774) | **0.017468** | 0.141684 |  |  |
| *CIITA* (rs6498124) |  |  | **0.038894** | 0.510149 |
| *HLA-DMB* (rs1042337) |  |  | **0.001268** | 0.09924 |

**
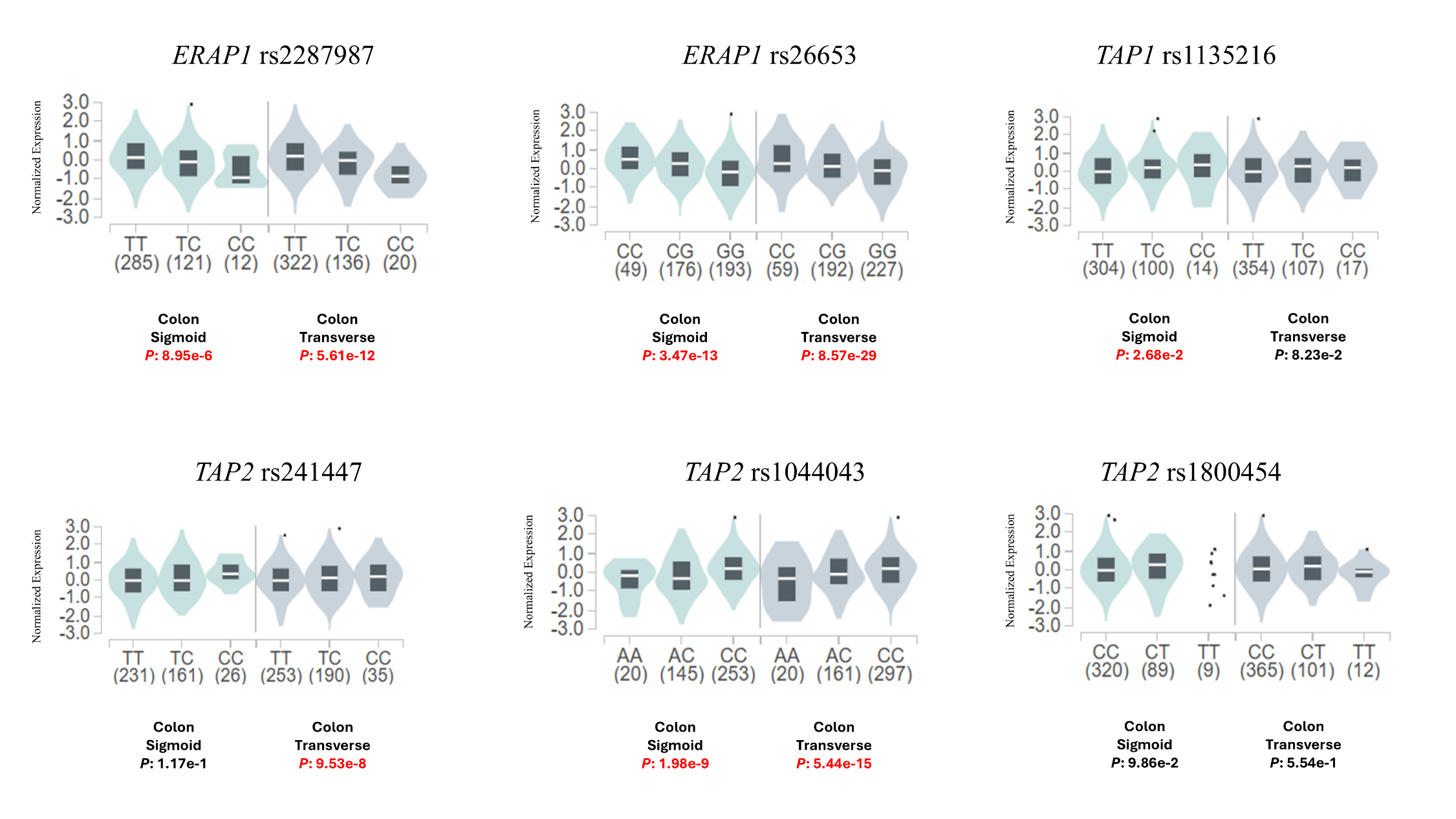
**

**Figure S1** Associations between selected SNPs and gene expression status in colon tissue from GTEx analysis (*ERAP1* rs2287987, *ERAP1* rs26653, *TAP1* rs1135216, *TAP2* rs241447, *TAP2* rs1044043, *TAP2* rs1800454)

**
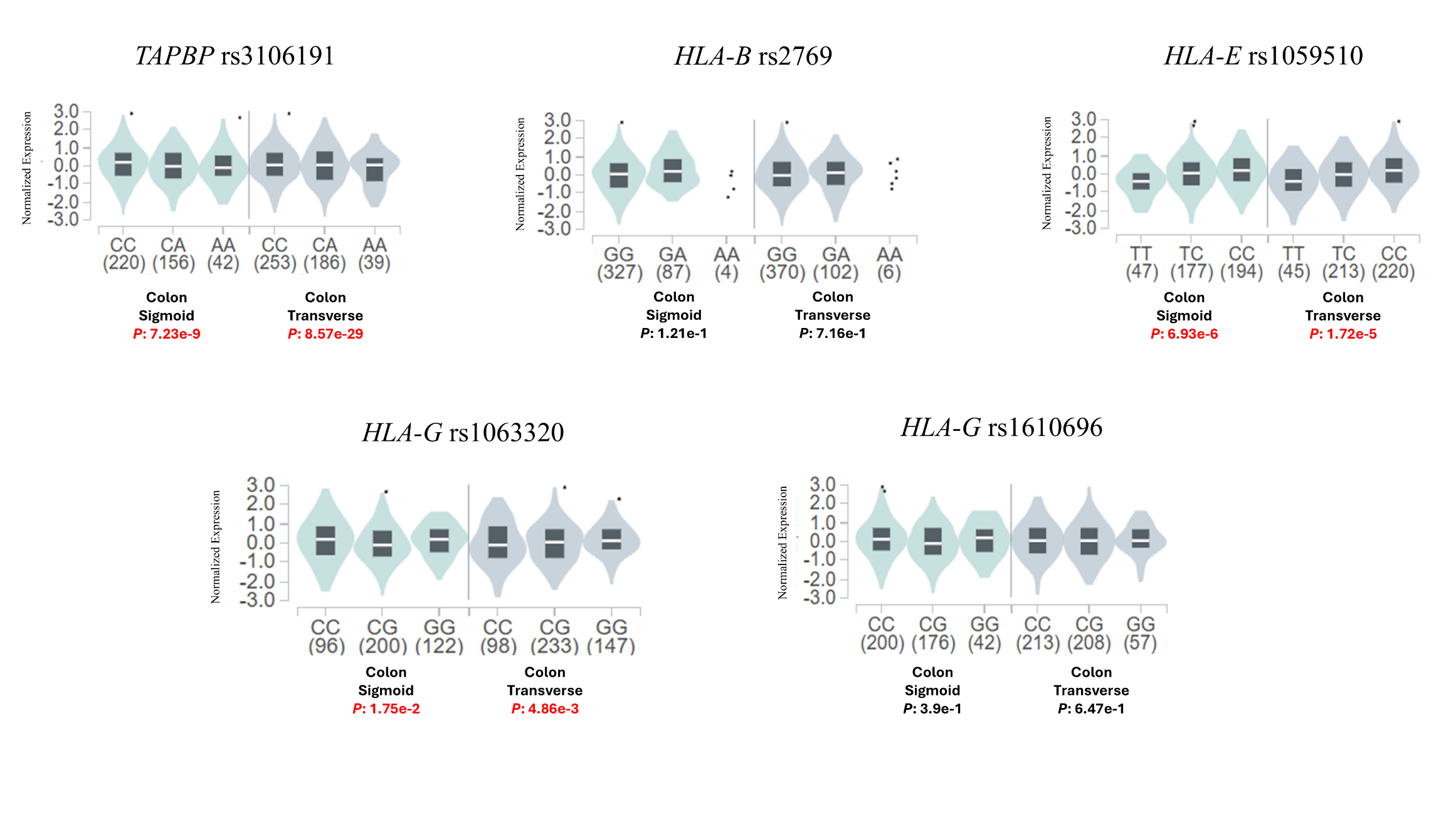
**

**Figure S2** Associations between selected SNPs and gene expression status in colon tissue from GTEx analysis (*TAPBP* rs3106191, *HLA-B* rs2769, *HLA-E* rs1059510, *HLA-G* rs1063320, *HLA-G* rs1610696)

**
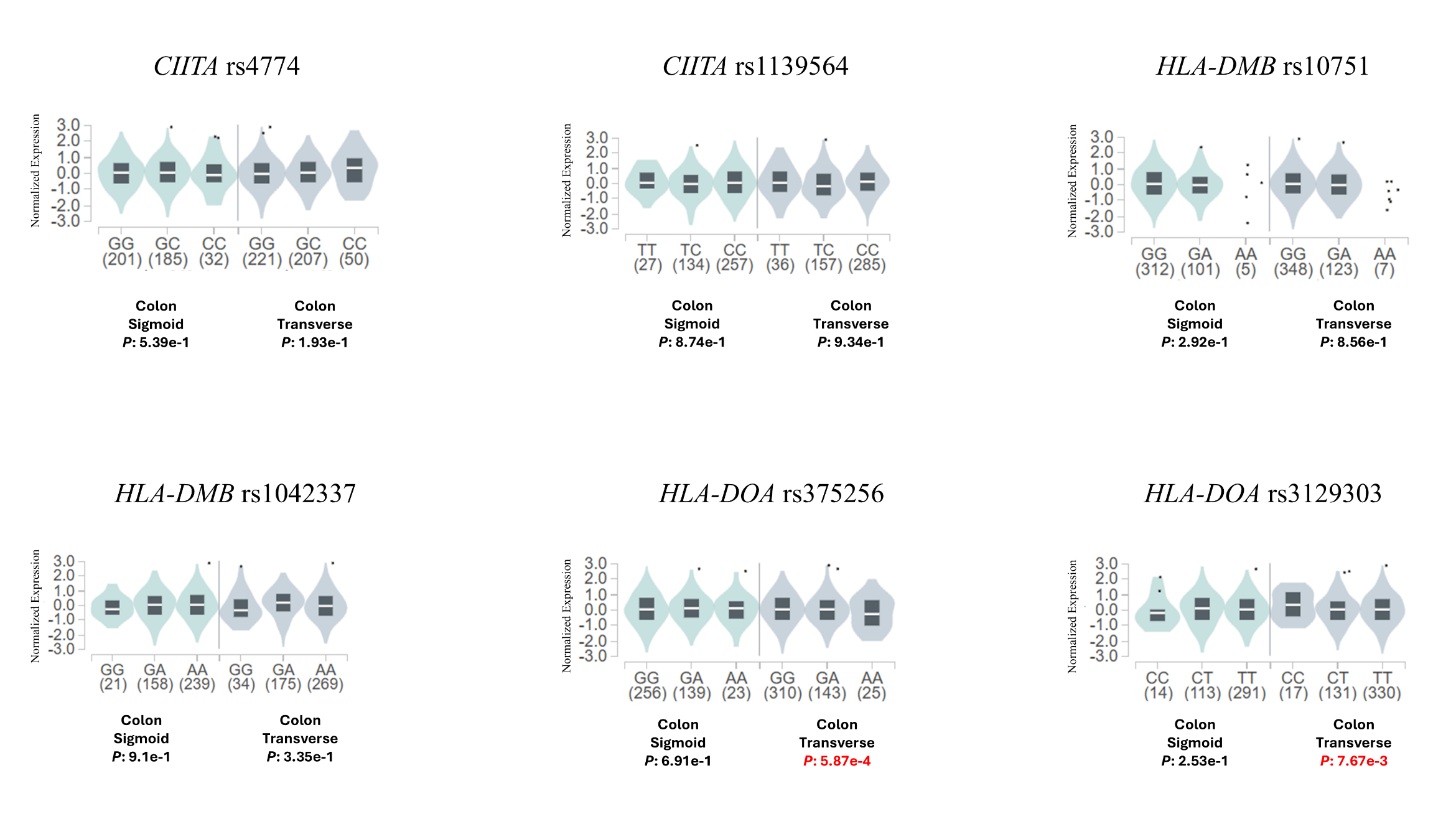
**

**Figure S3** Associations between selected SNPs and gene expression status in colon tissue from GTEx analysis (*CIITA* rs4774, *CIITA* rs1139564, *HLA-DMB* rs10751, *HLA-DMB* rs1042337, *HLA-DOA* rs375356, *HLA-DOA* rs3129303)

**
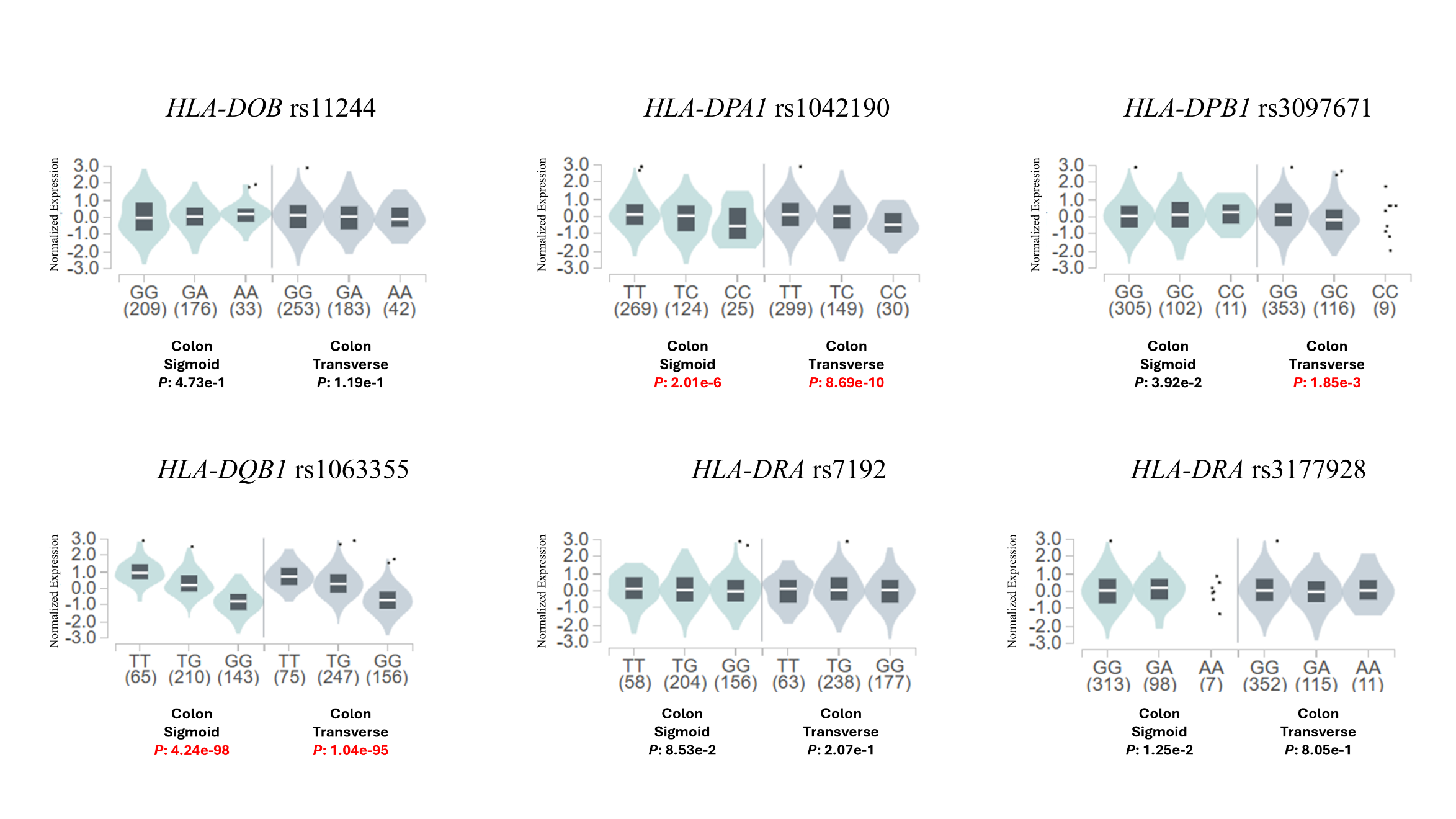
**

**Figure S4** Associations between selected SNPs and gene expression status in colon tissue from GTEx analysis (*HLA-DOB* rs11244, *HLA-DPA1* rs1042190, *HLA-DPB1* rs3097671, *HLA-DQB1* rs1063355, *HLA-DRA* rs7192, *HLA-DRA* rs3177928)
